# Quantitative neuropathology: an update on automated methodologies and implications for large scale cohorts

**DOI:** 10.1007/s00702-017-1702-2

**Published:** 2017-03-06

**Authors:** Lauren Walker, Kirsty E. McAleese, Mary Johnson, Ahmad A. Khundakar, Daniel Erskine, Alan J. Thomas, Ian G. McKeith, Johannes Attems

**Affiliations:** Institute of Neuroscience, Newcastle University, Campus for Ageing and Vitality, Newcastle upon Tyne, NE4 5PL UK

**Keywords:** Tissue microarray, Quantification, Alzheimer’s disease, Lewy body disease

## Abstract

A tissue microarray (TMA) has previously been developed for use in assessment of neurodegenerative diseases. We investigated the variation of pathology loads in semi-quantitative score categories and how pathology load related to disease progression. Post-mortem tissue from 146 cases were used; Alzheimer’s disease (AD) (*n* = 36), Lewy body disease (LBD) (*n* = 56), mixed AD/dementia with Lewy bodies (*n* = 14) and controls (*n* = 40). TMA blocks (one per case) were constructed using tissue cores from 15 brain regions including cortical and subcortical regions. TMA tissue sections were stained for hyperphosphorylated tau (HP-_T_), β amyloid and α-synuclein (αsyn), and quantified using an automated image analysis system. Cases classified as Braak stage VI displayed a wide variation in HP-_T_ pathology in the entorhinal cortex (interquartile range 4.13–44.03%). The interquartile range for β amyloid in frontal cortex in cases classified as Thal phase 5 was 6.75–17.03% and for αsyn in the cingulate in cases classified as McKeith neocortical LBD was 0.04–0.58%. In AD and control cases, HP-_T_ load predicted the Braak stage (*p* < 0.001), β amyloid load predicted Thal phase (*p* < 0.001) and αsyn load in LBD cases predicted McKeith type of LBD (*p* < 0.001). Quantitative data from TMA assessment highlight the range in pathological load across cases classified with ‘severe’ pathology and is beneficial to further elucidate the heterogeneity of neurodegenerative diseases. Quantifying pathology in multiple brain regions may allow identification of novel clinico-pathological phenotypes for the improvement of *intra vitam* stratification of clinical cohorts according to underlying pathologies.

## Introduction

Clinico-pathological correlative studies in dementia research have provided significant contributions into understanding how pathological protein aggregations in the brain correspond to the clinical manifestation of dementia. Seminal studies have demonstrated features of senile plaque accumulation and neurofibrillary tangle formation are essential to the neuropathological diagnosis of Alzheimer’s disease (AD) (Tomlinson et al. 1970) and more specifically emphasizing the importance of dystrophic neurites positive for hyperphosphorylated tau in neuritic plaques in AD cases compared to controls (Dickson et al. 1988; Probst et al. 1989; Arai et al. 1990). However, as patients were historically dichotomized as AD or normally aged controls, these studies lacked detail required to track disease progression. Later studies utilized semi-quantitative staging systems (based on 4 and 5-tiered staging scales) for diagnostics, and correlated pathological load against clinical measures of cognitive impairment (Braak and Braak 1991; Mirra et al. 1991; McKeith et al. 1996; Braak et al. 2003), giving further insight into the relative contribution of each pathology to clinical phenotype.

Neurodegenerative diseases by nature can be heterogeneous, and not all can be classified using current diagnostic criteria (Beach et al. 2009; Nelson et al. 2010), with semi-quantitative grades masking subtle differences in pathological burden. However, the recent advances in quantitative automated image analysis technologies have enabled the assessment of large scale cohorts and offers accurate and reproducible methods that can be implemented by researchers with varying degrees of neuropathology experience (Neltner et al. 2012; Attems et al. 2014). Using such technologies, differences in cases that classify as having ‘severe’ pathology have been reported; Murray and colleagues identified three distinct clinico-pathological phenotypes of AD (all Braak stage > IV), which have subsequently been predicted *intra vitam* by MRI (Whitwell et al. 2012), demonstrating direct translational impact of quantitative neuropathological data. In cases fulfilling neuropathological criteria for mixed dementia [AD and limbic/neocortical (LBD)] with different clinical phenotypes (i.e., AD or DLB), we found differences in the pathological burden and topographical distribution of hyperphosphorylated tau (HP-_T_), β amyloid and α-synuclein (α-syn) loads between clinical AD and DLB which were not detected using semi-quantitative criteria (Walker et al. 2015).

Clinico-pathological studies and in particular those that use quantitative methodologies are very labor intensive, which may limit the number of cases included in cohorts. As such, current studies are either: (1) very comprehensive, in which numerous brain regions are included but have relatively small sample sizes (Arriagada et al. 1992; Molano et al. 2010), (2) have a reasonable cohort size but include fewer brain regions included in the study (McKee et al. 1991; Kazee et al. 1993; Bartoo et al. 1997; Kovari et al. 2003), or (3) include large cohort sizes but pathology is assessed using neuropathological staging criteria or semi-quantitative scales which are not as time consuming (Jellinger 2006; Kovari et al. 2014). To address these limitations and to tease out discreet clinico-pathological phenotypes, it seems necessary for future studies to have large cohorts with numerous brain regions quantitatively assessed for multiple pathological lesions.

Tissue microarray (TMA) is a technique most commonly employed in tumor studies, which allows a large number of samples from individual cases to be relocated into a single block suitable for high throughput analysis (Kononen et al. 1998; Bubendorf et al. 2001), and has previously been employed to investigate white matter disease in a small sample of AD cases, highlighting its potential use in dementia research (Sjobeck et al. 2003). Here, we describe the application of TMA methodology to assess 15 anatomically distinct brain regions (40 samples in total) from any given case. In addition, we report on initial results from 146 cases (AD, LBD, mixed AD/LBD and controls) that have undergone TMA analysis for common neurodegenerative pathologies and illustrate a huge variation in pathology burden, in particular those classified as having ‘severe’ pathology by current diagnostic criteria (Thal et al. 2002; McKeith et al. 2005; Braak et al. 2006; Alafuzoff et al. 2008).

## Materials and methods

### Tissue preparation and neuropathological diagnosis

Brain tissue from 146 donors (mean age 79.91, SE ±0.72 years; male 89; female 57; AD 36; LBD (inclusive of DLB, PDD and PD) 56; mixed AD/DLB 14 and non-demented controls 40 (Table 1)], was obtained from Newcastle Brain Tissue Resource (NBTR) as part of a consecutive case series in accordance with the approval of the joint Ethics Committee of Newcastle and North Tyneside Health Authority and following NBTR brain banking procedures. During life, patients underwent clinical assessments including Mini-mental state examination (MMSE) (Folstein et al. 1975) by board certified Old Age Psychiatrists or Neurologists and clinical diagnoses were reviewed by AJT and IGM post-mortem, blinded to neuropathological diagnosis and checked against relevant standard international clinical criteria (McKhann et al. 1984, 2011; McKeith et al. 2005; Emre et al. 2007).

**Table 1:** Patient demographics

	AD	LBD	Mixed AD/DLB	Control
Case (*n*)	36	56	14	40
Age at death (mean, ± SE)	83.17 (1.32)	78.1	77.57 (1.50)	80.30 (1.86)
% Female	50	25	28.6	52.5
Braak NFT stage (Braak et al. 2006)	Stage 4 *n* = 1 Stage 5 *n* = 4 Stage 6 *n* = 31	Stage 0 *n* = 2 Stage 1 *n* = 7 Stage *n* = 14 Stage 3 *n* = 24 Stage 4 *n* = 9	Stage 5 *n* = 4 Stage 6 *n* = 10	Stage 0 *n* = 6 Stage 1 *n* = 8 Stage 2 *n* = 15 Stage 3 *n* = 9 Stage 4 *n* = 2
Thal phase (Thal et al. 2002)	Phase 4 *n* = 4 Phase 5 *n* = 32	Phase 0 *n* = 6 Phase 1 *n* = 3 Phase 2 *n* = 1 Phase 3 *n* = 6 Phase 4 *n* = 6 Phase 5 *n* = 4 NA *n* = 30	Phase 4 *n* = 1 Phase 5 *n* = 13	Phase 0 *n* = 13 Phase 1 *n* = 10 Phase 2 *n* = 6 Phase 3 *n* = 5 Phase 4 *n* = 1 Phase 5 *n* = 3 NA *n* = 2
CERAD (Mirra et al. 1991)	B *n* = 1 C *n* = 35	Negative *n* = 31 A *n* = 14 B *n* = 11	C *n* = 14	Negative *n* = 35 A *n* = 2 B *n* = 3
McKeith criteria (McKeith et al. 2005)	Negative *n* = 36	Limbic *n* = 13 Neocortical *n* = 43	Limbic *n* = 1 Neocortical *n* = 13	Negative *n* = 38 Brainstem *n* = 1 Amygdala predominant *n* = 1
MMSE (Folstein et al. 1975)	8.45 (1.81)	15.85 (1.82)	12.38 (3.34)	25.84 (1.53)

*AD* Alzheimer’s disease, *LBD* Lewy body disease, *n* number, *NFT* neurofibrillary tangle, *NA* not available, *CERAD* consortium to establish a registry for Alzheimer’s disease, *MMSE* mini-mental state examination

At autopsy the right hemisphere, brainstem and cerebellum were immersion fixed in 4% buffered aqueous formaldehyde for 4–6 weeks. Following fixation, the right hemisphere was dissected in coronal planes approximately 0.7 cm intervals and subjected to standard macroscopic examination, and brain regions required to determine the neuropathological diagnosis were sub-dissected and processed through increasing concentrations of alcohol then chloroform to paraffin wax. Subsequently, all brains underwent standard neuropathological assessment using internationally accepted criteria including neuritic Braak stages (Alafuzoff et al. 2008), Thal amyloid phases (Thal et al. 2002), CERAD scores (Mirra et al. 1991), NIA-AA scores (Montine et al. 2012) and McKeith criteria (McKeith et al. 2005).

### TMA construction

**Fig. 1:**
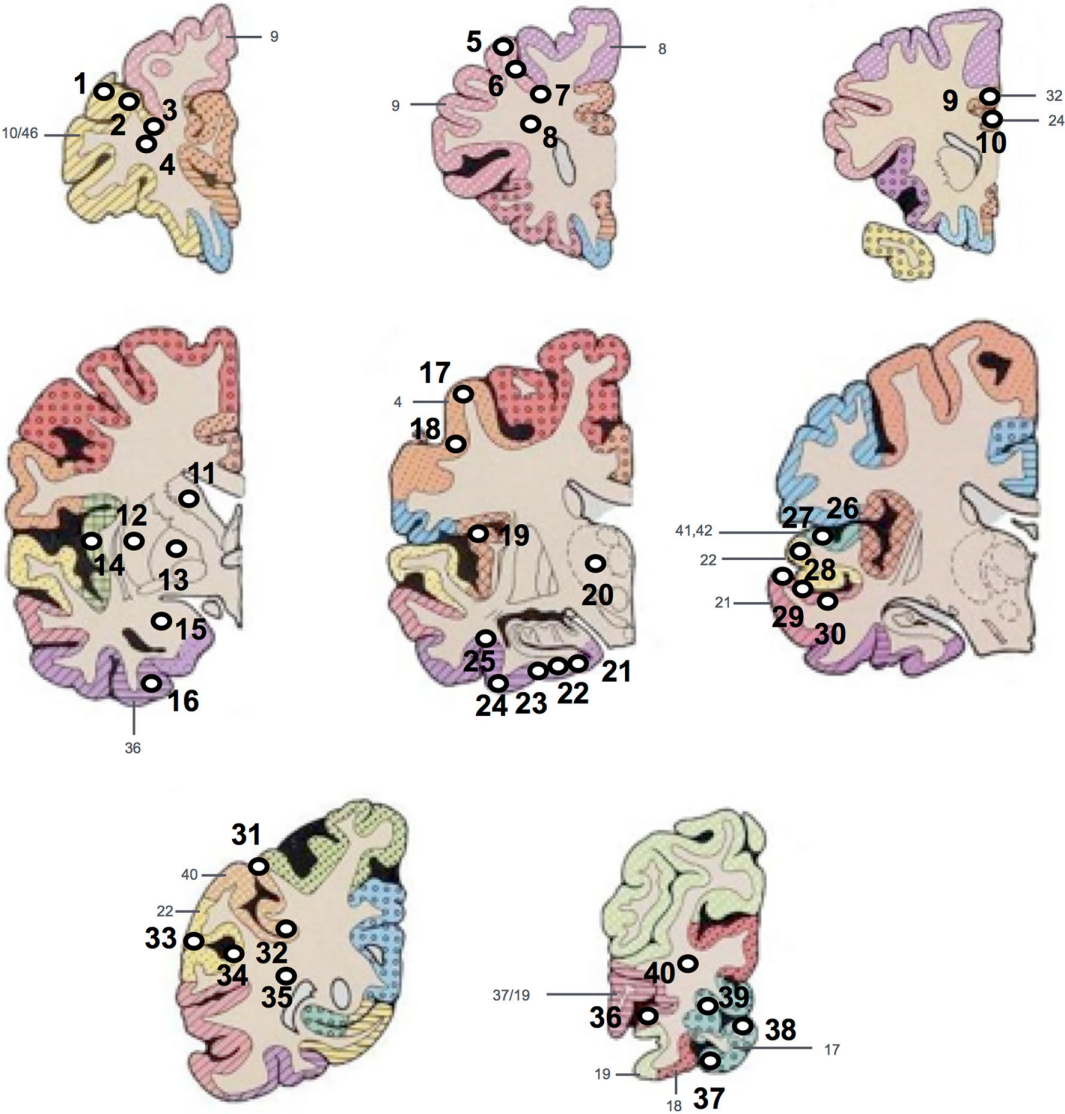
Diagram illustrating the locations where each of the tissue micro array (TMA) tissue cores were extracted from each diagnostic tissue block. Tissue cores 1–4 were taken from the pre-frontal cortex, 5–8 from mid-frontal cortex, 9 and 10 from the cingulate cortex, caudate, putamen, external globus pallidus, amygdala and insular cortex (11–16 + 19), 17 and 18 from motor cortex, thalamus (20), 21–25 from entorhinal cortex, 26–30 from temporal cortex, 31–35 from parietal cortex and 36–40 from occipital cortex. *White circles* and *black numbers* represent the tissue cores with numeric label. *Gray numbers* and *color coding represent* Brodmann areas Adapted from (Perry and Oakley 1993)

Each case was then sampled to compose a TMA block. Areas that were sampled for the TMA were taken from paraffin embedded (donor) blocks containing: pre-frontal cortex [Brodmann area 9 (BA), 10/46], mid-frontal cortex, (BA8, 9), cingulate gyrus (BA24, 32), caudate, putamen, external globus pallidus, amygdala, insular cortex, motor cortex (BA4), thalamus, entorhinal cortex, temporal cortex (BA21, 22, 41/42), parietal cortex (BA22, 40) and occipital cortex (BA17, 18, 19, 19/37) (Fig. 1).

**Fig. 2:**
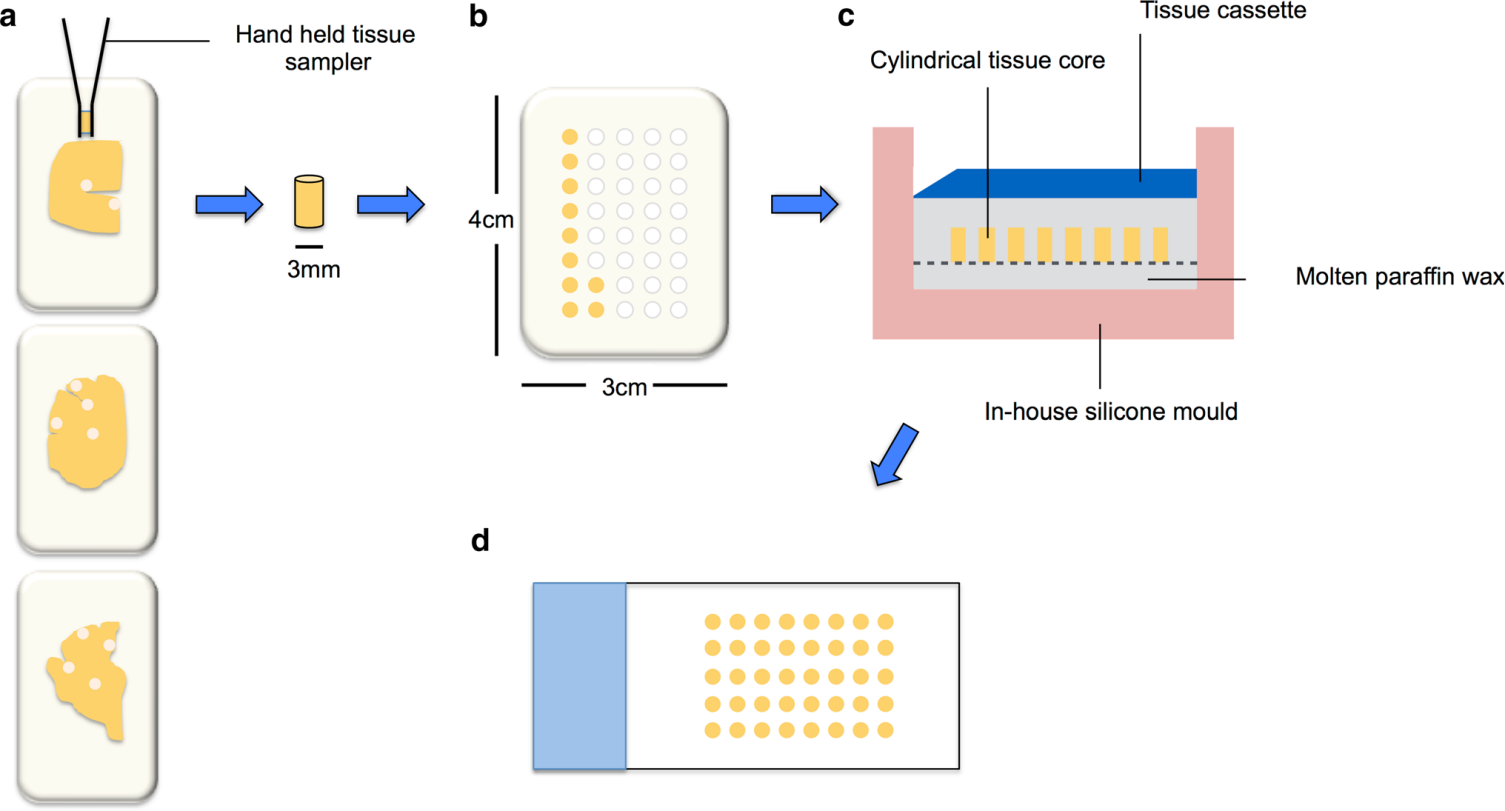
Schematic illustrating the production of the Tissue Microarray (TMA) block. 3 mm cylindrical tissue cores are taken from pre-defined positions from fixed paraffin embedded donor blocks using a hand held tissue sampler (**a**). Each core is then inserted into the correct hole in a pre-made recipient block in numerical order (**b**). The completed recipient block is then placed face down in a mould specifically made to fit the block with 2–4 ml of molten wax in the bottom, and left to anneal (**c**). TMA sections are then cut at 6 μm and mounted onto glass slides (**d**)

Fixed paraffin donor blocks were warmed for 1 h at 37 °C to aid tissue removal. 3 mm cylindrical tissue cores were taken from predefined positions using a hand held tissue sampler (Tissue-Tek^®^ Quick-Ray™ TMA system, Sakura, CA, USA). The tip of the hand held punch was inserted at the correct position through the depth of the tissue in the donor block and removed along with the cylindrical tissue core (Fig. 2a). Each tissue core was then inserted into the correct ‘hole’ in a single, regular sized pre-made recipient TMA paraffin block (4 cm × 3 cm—made to perfectly match the Tissue-Tek^®^ Quick-Ray™ TMA system) in numerical order (Fig. 2b). If any donor blocks are missing, empty ‘holes’ in recipient block were filled with molten wax. Each of the punches was pushed securely into the recipient block by hand and the block incubated at 37 °C to reduce the wax–tissue interface when sectioning.

An in-house silicone mould, made specifically to fit the TMA recipient block, was preheated to 60 °C for 1 h and 2–4 ml of molten paraffin wax placed into the base. The recipient TMA block was placed tissue face down onto the molten wax and left for 5 min before a further 15 min incubation at 37 °C to anneal (Fig. 2c). The block was allowed to cool fully before the silicone mould was removed and excess wax removed. TMA sections were then cut and mounted onto glass slides (Fig. 2d) and immunohistochemically stained for HP-_T_ (AT8 clone, Innogenetics, Belgium), β amyloid (4G8 clone, Covance, UK) and α-syn (Leica, UK) as described previously (Walker et al. 2015).

### Image analysis

**Fig. 3:**
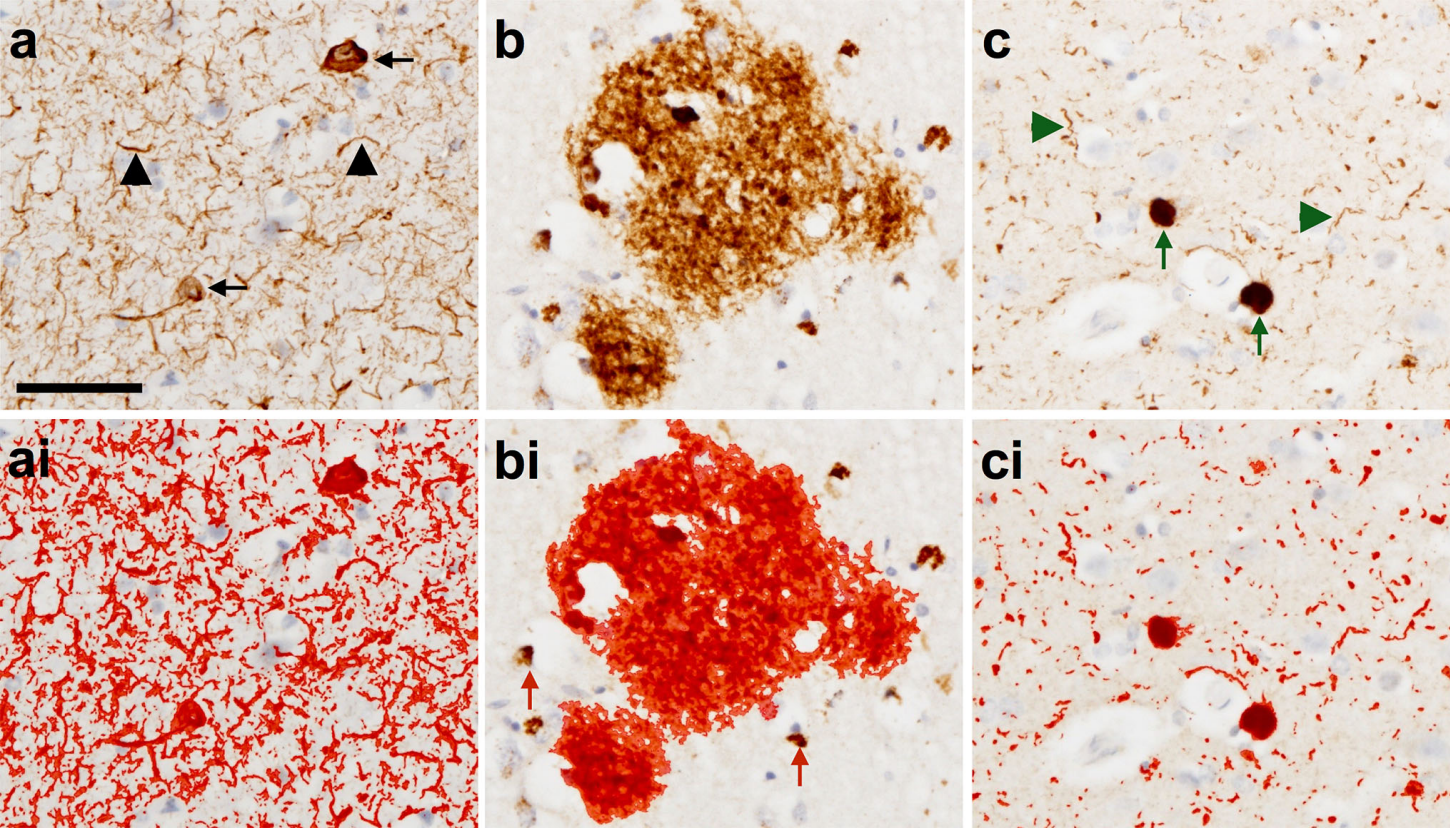
Photomicrographs illustrating immunohistochemically stained pathology (**a**–**c**) and the application of a standardized threshold designed to capture all immunopositive signals to be included in the quantitative analysis (*red outline* in **ai**–**ci**). Neurofibrillary tangles (*black arrow*) and neuropil threads (*black arrowhead*) are immunopositive for HP-_T_ (AT8 antibody) (**a**) and with threshold applied—*red outline* (**ai**). Plaques are immunopositive for β amyloid (4G8 antibody) (**b**) and with the threshold applied—*red outline* (**bi**). Intracellular amyloid precursor protein is also immunopositive using 4G8 antibody and is excluded from the quantitative analysis using a size restriction threshold (*red arrows*) (**bi**). Lewy bodies (*green arrows*) and Lewy neurites (*green arrowheads*) are immunopositive for α-syn (α-syn antibody) (**c**) and with threshold applied—*red outline* (**ci**). *Scale bar* in a represents 50 μm and is valid for all images

TMA slides were analyzed using an automated system consisting of a Nikon Eclipse 90i microscope, DsFi1 camera and NIS Elements software v 3.0 (Nikon). Sections were placed onto the microscope in the correct orientation to position tissue core 1 in the top left field of view. To capture images of each of the 40 tissue cores on the TMA slide, the microscope was positioned in the center of the first tissue core at 20× magnification for guidance and brought into focus at 100× magnification. The co-ordinates of this first tissue core were then mapped using a macro designed to take large images comprised of 3 × 3 small images measuring 1.7 mm^2^. The microscope was then positioned over the center of the second tissue core and the process repeated until the positions of all 40 tissue cores had been mapped. If any of the tissue cores were missing or too damaged to be included in the analysis the tissue core number was noted and this was factored into the analysis. Once all 40 tissue cores were mapped, the microscope was directed to the co-ordinates of the first tissue core and the images of all tissue cores were automatically taken in sequence. Regions of interest (ROI) were applied to individual images if necessary to exclude any white matter or abnormalities in the tissue (e.g., folded tissue or tears). Restriction threshold was applied to capture all immunopositive signals. The measurement of immunopositivity and subsequent calculation of the percentage area covered by immunopositivity was performed using an automated methodology. Red, Green and Blue (RGB) thresholds that determine the pixels that are included in the binary layer used for measurement were standardized separately for each AT8, 4G8 and α-syn immunopositivity and thresholds were set at a level that was reached by immunopositive pathological structures only (except *APP*; see below). RGB intensity values are measured on a scale between 0 and 255 (see NIS elements version 3.0, user guide, 2008, Nikon, Surrey UK) and were set as follows; AT8: R25–170, G27–156, B11–126; 4G8: R50–180, G20–168, B8–139, α-syn: R15–161, G7–139, B4–133 (Fig. 3). Thereby, unspecific background staining did not reach the threshold and was not included into the measurement. In addition to RGB thresholds, we set a restriction threshold for the assessment of 4G8 immunopositivity that excluded the measurement of immunopositive signals of a size below 100 μm^2^; this was necessary to ensure that physiological, cellular *APP* that is stained with 4G8 antibody was not included in the measurement. Of note, the exclusion of areas below 100 μm^2^ implies that pathological β amyloid depositions of less than 100 μm^2^ were not included into the measurement. However, diffuse β amyloid depositions and β amyloid plaques are typically larger than 100 μm^2^ (Duyckaerts et al. 2009). Of note, only immunoreactive neurones harboring HP-_T_ positive NFTs and NTs were quantified in sections stained with AT8 antibody. Glial HP-_T_ pathology such as for example seen in progressive supranuclear palsy (Dickson et al. 2007), corticobasal degeneration (Dickson et al. 2002), and aging-related tau-astrogliopathy (ARTAG) (Kovacs et al. 2016) was identified on visual inspection and excluded when quantifying pathology as part of TMA. In addition, TMA punches that were devoid of β amyloid plaques but had severe cerebral amyloid angiopathy on 4G8 stained sections were disregarded in quantitative analysis. Percentage area of the tissue covered by immunopositivity was subsequently calculated and for brain regions that had more than one tissue core, mean values were calculated.

### Statistical analysis

Statistical analysis was conducted using the Statistical Package for Social Sciences (SPSS v 22, IBM). Data was tested for normality using Kolmogorov–Smirnov test followed by visual inspection of variable histograms. Kruskal–Wallis was used to determine overall differences between groups and a non-parametric *t* test (Mann–Whitney *U*) to assess post hoc differences between individual groups. Spearman’s correlation coefficients (two tailed) were used to assess associations between pathology load with pathological stages and MMSE scores. Exploratory linear regression analyses were conducted to investigate predictors of disease progression and cognitive decline.

## Results

### Neocortical pathology increases with disease progression

**Fig. 4:**
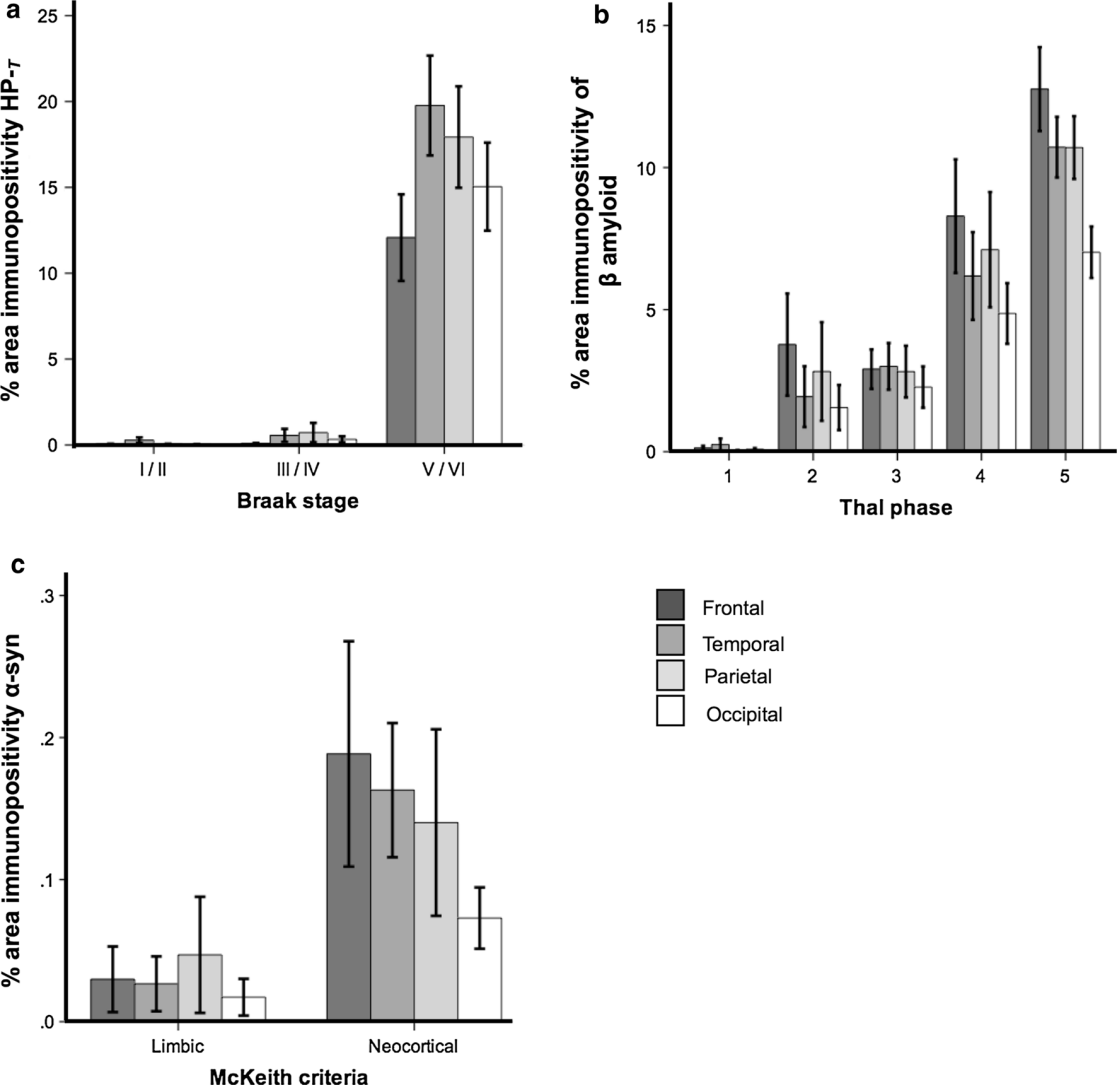
Mean neocortical hyperphosphorylated tau (HP-_T_), β amyloid and α-synuclein (α-syn) load significantly increases in line with neurofibrillary tangle (NFT) Braak stages, Thal phases and McKeith criteria. For mean values and statistics, see Table 2

Mean neocortical HP-_T_ load increased significantly (*p* < 0.001) with increasing NFT Braak stage in all neocortical regions (Fig. 4a), as did neocortical β amyloid (*p* < 0.001) in line with increasing Thal phases (Fig. 4b). Although α-syn load was higher in all regions classified as neocortical LBD compared to limbic LBD, only the increase in temporal lobe α-syn load was significant (*p* < 0.05) (Fig. 4c). For post hoc statistics see Table 2.

**Table 2:** Neocortical pathology burden of hyperphosphorylated tau, β amyloid, and α-synuclein and neuropathological criteria

Braak NFT stage				
	I/II (*n* = 47)	III/IV (*n* = 42)	V/VI (*n* = 57)	Statistic^*^
HP-T load				
Frontal % (± SE)	0.07 (0.01)	0.10 (0.02)	12.29 (2.48)	*p* < 0.001^a^
Temporal % (± SE)	0.26 (0.14)	0.60 (0.33)	20.58 (2.85)	*p* < 0.001^b^
Parietal % (± SE)	0.06 (0.02)	0.70 (0.52)	18.07 (2.90)	*p* < 0.001^c^
Occipital % (± SE)	0.04 (0.01)	0.37 (0.17)	15.04 (2.56)	*p* < 0.001^d^

Thal Aβ phase						
	1 (*n* = 13)	2 (*n* = 7)	3 (*n* = 11)	4 (*n* = 5)	5 (*n* = 45)	Statistic^*^
Aβ load						
Frontal % (± SE)	0.13 (0.07)	3.80 (1.80)	2.91 (0.69)	8.29 (1.20)	12.80 (1.44)	*p* < 0.001^e^
Temporal % (± SE)	0.26 (0.20)	1.94 (1.07)	3.00 (0.82)	5.97 (1.42)	10.76 (1.04)	*p* < 0.001^f^
Parietal % (± SE)	0.09 (0.44)	2.82 (1.73)	2.82 (0.91)	7.37 (1.87)	10.63 (1.08)	*p* < 0.001^g^
Occipital % (± SE)	0.17 (0.09)	1.55 (0.79)	2.27 (0.73)	4.86 (1.07)	7.02 (5.99)	*p* < 0.001^h^

McKeith criteria			
	Limbic (*n* = 7)	Neocortical (*n* = 36)	Statistic^**^
α-Syn load			
Frontal % (± SE)	0.06 (0.04)	0.20 (0.07)	ns
Temporal % (± SE)	0.04 (0.02)	0.17 (0.04)	*p* < 0.05
Parietal % (± SE)	0.05 (0.04)	0.16 (0.06)	ns
Occipital % (± SE)	0.02 (0.01)	0.09 (0.03)	ns

*NFT* neurofibrillary tangle, *n* number, *HP-T* hyperphosphorylated tau, *SE* standard error, *Aβ* amyloid beta, *α-syn* alpha-synuclein, *ns* not significant

* Kruskal–Wallis test, Pairwise post hoc Mann–Whitney *U* tests

** Mann Whitney *U* test

^a^I/II < III/IV (*p* < 0.01), I/II < V/VI and III/IV < V/VI (both *p* < 0.001)

^b^I/II < III/IV (*p* < 0.01), I/II < V/VI and III/IV < V/VI (both *p* < 0.001)

^c^I/II < III/IV (*p* < 0.01), I/II < V/VI and III/IV < V/VI (both *p* < 0.001)

^d^I/II < III/IV (*p* < 0.01), I/II < V/VI and III/IV < V/VI (both *p* < 0.001)

^e^1 < 3, 1 < 4, 1 < 5, 3 < 5 (all *p* < 0.001) and 2 < 5, 3 < 4 (*p* < 0.01)

^f^1 < 3, 1 < 4, 1 < 5, 2 < 5 3 < 5 (all *p* < 0.001) and 4 < 5 (*p* < 0.05)

^g^1 < 3, 1 < 4, 1 < 5, 3 < 5 (all *p* < 0.001) and 2 < 3 (*p* < 0.01)

^h^1 < 3, 1 < 4, 1 < 5 (all *p* < 0.001), 3 < 5 (*p* < 0.01) and 2 < 4 (*p* < 0.05)

### Variation of pathology in ‘severe’ semi-quantitative grades

**Fig. 5:**
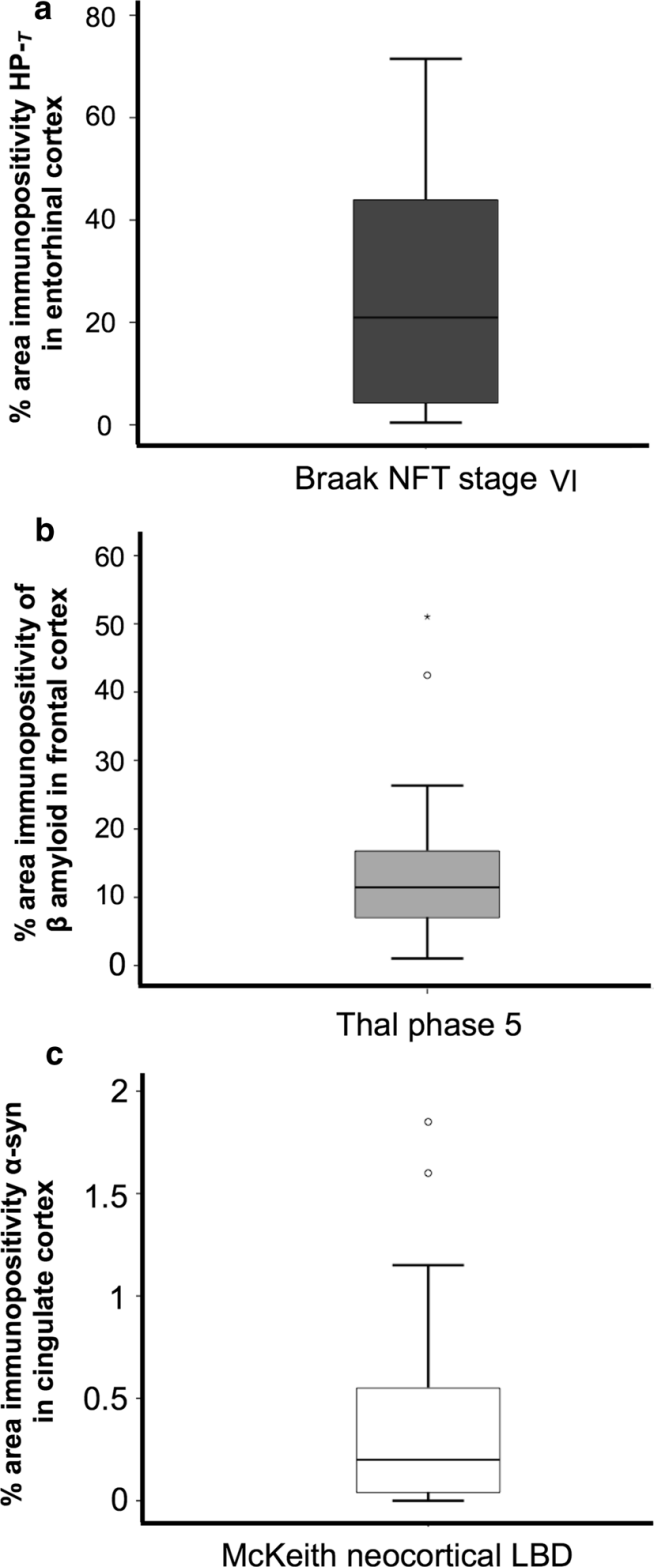
Cases classified as having ‘severe’ pathology display a large variation in pathology load. **a** Hyperphosphorylated tau (HP-_T_) load in the entorhinal cortex in cases classified as neuritic Braak stage VI. **b** β amyloid load in the frontal cortex in cases classified as Thal phase 5 and **c** α-Synuclein (α-syn) load in the cingulate cortex in Lewy body disease (LBD) cases that are classified as McKeith neocortical LBD

Of the cases classified as Braak stage VI, the median HP-_T_ load in the entorhinal cortex was 20.96%; however, the range of HP-_T_ load was extensive (minimum 0.43%, maximum 71.48% and interquartile range 4.13–44.03%) (Fig. 5a). In cases classified as Thal phase 5 the median β amyloid load in the frontal cortex was 11.45% (minimum 1.03%, maximum 51.03% and interquartile range 6.75–17.03%) (Fig. 5b). Cases that fulfilled McKeith criteria for neocortical LBD harbored a median α-syn load of 0.2% (minimum 0.001%, maximum 1.85%, interquartile range 0.04–0.58%) in the cingulate cortex (Fig. 5c).

### Associations between pathology load with staging criteria and cognitive decline

**Fig. 6:**
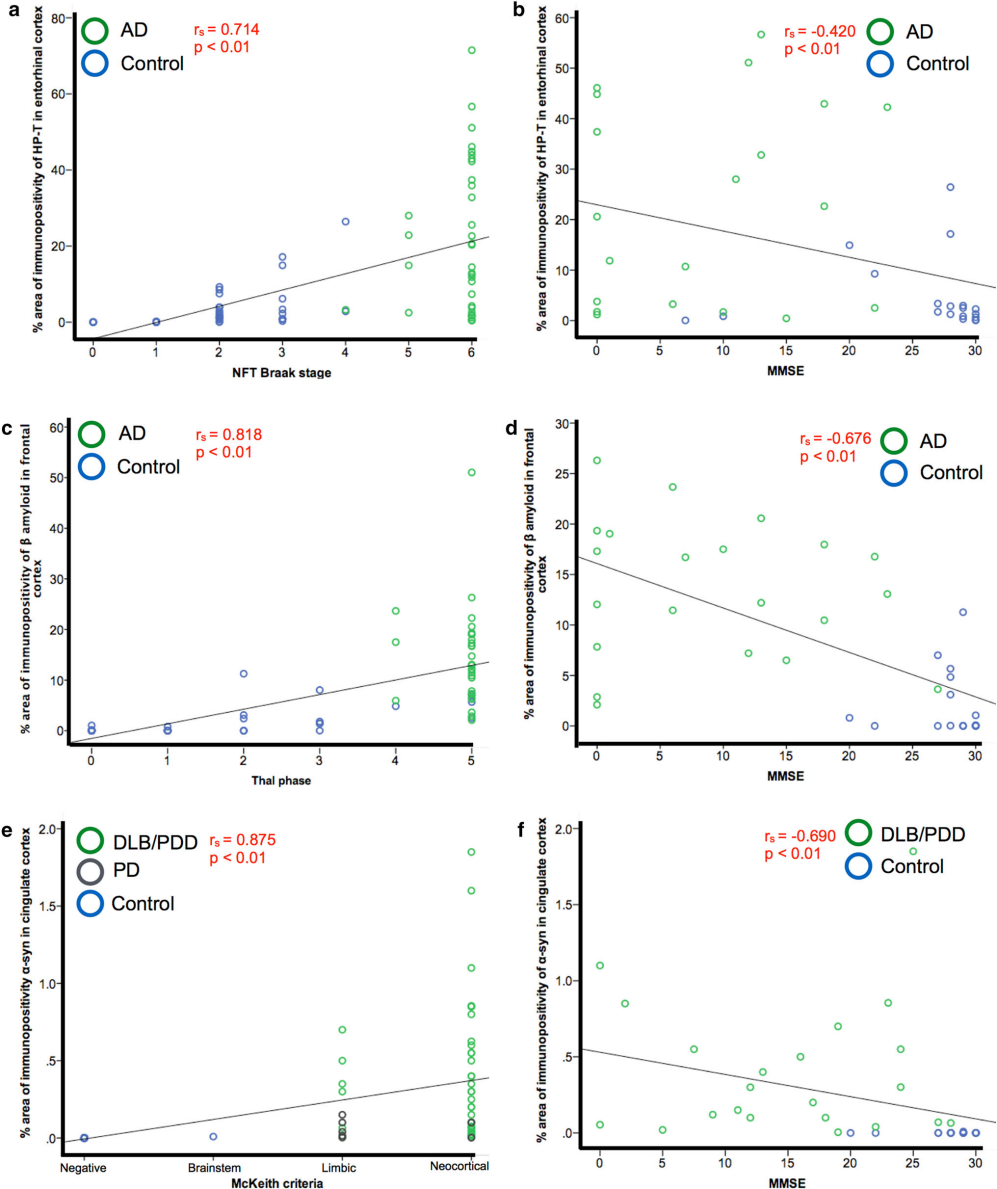
Entorhinal cortex, frontal cortex and cingulate cortex were selected as regions affected early in disease progression to investigate the accumulation of pathology during increasing stages of disease progression and the impact on cognitive decline. Hyperphosphorylated tau (HP-_T_) load in the entorhinal cortex positively correlated with neurofibrillary tangle (NFT) Braak stage (**a**) and negatively correlated with MMSE score (**b**) in AD and control cases. β amyloid load in the frontal cortex positively correlated with Thal phase (**c**) and negatively correlated with MMSE score (**d**) in AD and control cases. α-Synuclein (α-syn) load in the cingulate cortex positively correlated with McKeith criteria in dementia with Lewy body (DLB), Parkinson’s disease dementia (PDD) Parkinson’s disease (PD) and control cases (**e**) and negatively correlated with with MMSE scores in DLB, PDD and controls (**f**) (PD cases were not included as no MMSE scores were available for these cases)

We investigated whether HP-_T_ load in the entorhinal cortex was associated with disease progression as measured by NFT Braak stage and cognitive decline as measured by MMSE. AD and control cases were selected to display a full range of NFT Braak stages. HP-_T_ load positively correlated with NFT Braak stage (*r*_s_ = 0.714, *p* < 0.01) (Fig. 6a). In cases where MMSE scores were available (*n* = 37), HP-_T_ load negatively correlated with MMSE score (*r*_s_ = −0.420, *p* < 0.01) (Fig. 6b). In AD and control cases β amyloid load in the frontal cortex positively correlated with Thal phase (*r*_s_ = 0.818, *p* < 0.01) (Fig. 6c) and frontal β amyloid load negatively correlated with MMSE score (*r*_s_ = 0.676, *p* < 0.01) (Fig. 6d). Whilst in LBD and control cases, α-syn load in the cingulate positively correlated with disease progression as measured by McKeith criteria (*r*_s_ = 0.875, *p* < 0.01) (Fig. 6e). In cases where MMSE scores were available (*n* = 39) α-syn load in the cingulate negatively correlated with MMSE score (*r*_s_ = 0.690, *p* < 0.01) (Fig. 6f).

### Pathology load predicts disease progression and cognitive decline

To address whether pathology load could predict disease progression and cognitive decline as measured by MMSE, we performed exploratory linear regression analyses in AD, LBD and control cases. HP-_T_ load in the entorhinal cortex predicted both NFT Braak stage (model *R* ^2^ = 0.324, *F*_(1)_ = 35.423, *p* < 0.001) and MMSE score (model *R* ^2^ = 0.116, *F*_(1)_ = 4.873, *p* < 0.05). In AD and control cases, β amyloid load in the frontal cortex predicted Thal phase (model *R* ^2^ = 0.421, *F*_(1)_ = 48.025, *p* < 0.001) and MMSE score (model *R* ^2^ = 0.413, *F*_(1)_ = 25.277, *p* < 0.001). Whilst in LBD and control cases α-syn load in the cingulate predicted disease progression as classified by McKeith criteria (model *R* ^2^ = 0.260, *F*_(1)_ = 43.959, *p* < 0.001) and MMSE score (model *R* ^2^ = 0.119, *F*_(1)_ = 4.983, *p* < 0.05).

## Discussion

Using a TMA-based methodology, we have developed an automated quantification technique capable of accurately assessing multiple pathological lesions in a range of neurodegenerative diseases, whilst the inclusion of 15 brain regions, represented by a total of 40 samples, provides a platform to study *post-mortem* tissue on a systems level in the neuropathology context. Data from 146 cases currently analyzed displayed a wide variation in pathology load in semi-quantitative grades in particular HP-_T_ load in cases classified as NFT Braak VI. This is in agreement with a previous study by Abner and colleagues, who observed an appreciable range in neurofibrillary pathology in neocortical regions in Braak stage VI cases, with the most severely affected cases displaying increased *ante-mortem* cognitive impairment (Abner et al. 2011). Such variations in pathology highlights the need for quantitative neuropathological assessment in future clinico-pathological studies, as Braak staging can mask severe variations in pathology severity and may account for some of the heterogeneity observed in neurodegenerative diseases in addition to hindering identification of discreet novel clinico-pathological phenotypes. Although the TMA methodology presented here allows the accurate assessment of pathological lesions in multiple brain regions, there is an inherent bias associated with this type of technique. It has been previously shown that densities of pathological protein aggregates (e.g., β amyloid) differs between gyri and sulci (Gentleman et al. 1992), and as such we have tried to limit anatomical bias by sampling gyri and sulci of each cortical region within the TMA block. Other brain regions incorporated into the TMA block, such as the striatum and thalamus, are more complex structures containing multiple nuclei, and whilst this technique is aimed at providing an overview of pathology present, we suggest a more comprehensive sampling protocol when investigating individual brain regions.

Previous studies have demonstrated that staging disease progression using standardized criteria is the most accurate correlate of the neurodegenerative process (Braak and Braak 1991; Bancher et al. 1996), however, here, we have demonstrated that HP-_T_, β amyloid and α-syn load in individual brain regions increases with, and is a predictor of increasing neuropathological stage. In addition we have shown that HP-_T_, β amyloid and α-syn loads in the entorhinal cortex, frontal cortex and cingulate cortex, respectively, are predictors of cognitive decline as measured by MMSE, which highlights the importance of multiple pathologies in neurodegeneration and this may have implications for future therapeutic design strategies. A recent study by Koss and colleagues found pre-fibrillar soluble forms of HP-_T_ and β amyloid are present early in the disease course and are closely linked to disease progression and cognitive impairment (Koss et al. 2016). Therefore, targeting pre-fibrillar forms of HP-_T_ and β amyloid or insoluble deposits in the early stage of disease before considerable accumulation may be a more effective treatment strategy. However, research into prion-like mechanisms involved in propagation of misfolded proteins in neurodegenerative diseases is gathering pace, with drug development targeted at halting the cell to cell transmission of toxic protein aggregates (for reviews see Frost and Diamond 2010; Hasegawa et al. 2016). It is, therefore, essential to address both the accumulation of pathological aggregations in a given region at an early stage and the potential spread to other regions when designing novel therapeutics.

The finding that pathology load in a given region is associated with cognitive decline prompts the question whether there is a pathological ‘threshold’ that is needed to be breached to elicit symptoms of cognitive impairment. A clinico-pathological study by Haroutunian and colleagues reported no difference in HP-_T_ load in subjects with ‘questionable dementia’ (CDR 0.5) and control subjects (CDR 0) (Haroutunian et al. 1999). However, the study utilized a semi-quantitative scoring system, which may not have been sensitive enough to detect subtle differences in pathology. Future studies using a high throughput quantitative methodology, such as presented here, may reveal a saturation point at which cognitive symptoms are clinically observed.

Although clinico-pathological studies have been paramount to research into neurodegenerative dementias, data from previous studies may be biased as they may have assessed hallmark pathologies of one neurodegenerative disease category only. It is apparent that neuropathological lesions associated with neurodegenerative dementias are not exclusive to single diseases (Jellinger 2007; Attems and Jellinger 2013) and co-existing pathologies confer a worse prognosis (Olichney et al. 1998; Serby et al. 2003; Kraybill et al. 2005), therefore the presence of co-morbid pathologies needs to be taken into account when conducting large scale studies. A recent large scale clinico-pathological correlative study by Irwin and colleagues investigated the effect of concomitant pathologies in a *post-mortem* cohort of cases with synucleinopathies. They report an increased severity of Lewy body pathology in addition to AD pathology (in particular HP-_T_ pathology) resulted in a shorter survival time and a shorter interval between motor symptoms to the onset of dementia (Irwin et al. 2017). They also suggest that future cohorts are stratified by their level of AD pathology in clinical trials for promising therapeutics targeting HP-_T_, β amyloid and α-syn. Our TMA methodology provides the appropriate platform to address the issues of co-morbid pathologies in neurodegenerative diseases, as it allows serial sections to be stained for multiple pathological lesions in a considerably shorter time frame than traditional quantification techniques using whole tissue sections. The construction of the TMA block and subsequent sectioning and staining can be completed in a day, whilst the quantification of each individually stained TMA slides can be completed in an hour, making TMA a reliable high throughput system. Assessment of neuropathological lesions can be reliably conducted by neuropathologists with a wealth of experience, such as Professor Kurt Jellinger (Paulus et al. 1992; Bancher et al. 1997). However, quantification techniques such as the one described in this study will allow researchers with considerably less experience to assess pathology loads in neurodegenerative diseases under the supervision of an experienced neuropathologist or researcher with an expertise in the human neuropathology of neurodegeneration. The purpose of this quantitative TMA technique is not to replace, but to complement diagnostic procedures, and add value to human *post-mortem* tissue donated to brain banks for research purposes. Data generated by this technique can be incorporated into multivariate models used for clinico-pathological studies.
